# Sociodemographic and Lifestyle Determinants of HIF-1α Response to Blood Donation and Hematopoietic Factors: Epidemiological and Public Health Perspectives from Voluntary Donors

**DOI:** 10.3390/epidemiologia7010009

**Published:** 2026-01-05

**Authors:** Svjetlana Gašparović Babić, Ivana Paver, Tomislav Rukavina, Lara Batičić

**Affiliations:** 1 Department of Public Health, Teaching Institute of Public Health of Primorje-Gorski Kotar County, 51000 Rijeka, Croatia; svjetlanagb@uniri.hr; 2 Department of Public Health, Faculty of Health Studies, University of Rijeka, 51000 Rijeka, Croatia; 3 Clinical Institute of Transfusion Medicine, Clinical Hospital Center Rijeka, 51000 Rijeka, Croatia; ivana.paver@kbc-rijeka.hr; 4 Department of Clinical Laboratory Diagnostics, Faculty of Medicine, University of Rijeka, 51000 Rijeka, Croatia; 5 Department of Social Medicine and Epidemiology, Faculty of Medicine, University of Rijeka, 51000 Rijeka, Croatia; tomislav.rukavina@uniri.hr; 6 Department of Clinical Microbiology, Teaching Institute of Public Health of Primorje-Gorski Kotar County, 51000 Rijeka, Croatia; 7 Department of Medical Chemistry, Biochemistry and Clinical Chemistry, Faculty of Medicine, University of Rijeka, 51000 Rijeka, Croatia

**Keywords:** blood donation, dietary patterns, epidemiology, hematopoietic factors, hypoxia-inducible factor 1-alpha, lifestyle habits

## Abstract

**Background/Objectives:** Blood donation is essential to health systems and represents a valuable epidemiological model for studying physiological adaptation to controlled blood loss. Regular blood donors constitute a distinct, health-screened population whose biological responses offer unique insight into mechanisms of resilience and key determinants of population health. Hypoxia-inducible factor 1-alpha (HIF-1α) is a key regulator of erythropoiesis and cellular response to hypoxia, and its modulation following blood donation may inform donor safety and the sustainability of blood donation programs. This study aimed to characterize the sociodemographic, lifestyle, and anthropometric profiles of blood donors in relation to hematopoietic biomarkers (vitamin B12 and folic acid) and to evaluate changes in serum HIF-1α concentration after donation, emphasizing the public health relevance of voluntary blood donation. **Methods:** A cross-sectional study was conducted among 324 voluntary blood donors (159 regular and 165 occasional). Serum HIF-1α was measured before and 30 min after donation, together with vitamin B12 and folic acid levels. Sociodemographic and lifestyle characteristics (physical activity, smoking, dietary habits) were collected through standardized questionnaires (EHIS-3, FFQ), and anthropometric parameters were assessed. **Results:** Regular donors were older and predominantly male, with comparable socioeconomic indicators between groups. Both regular and occasional donors showed favorable lifestyle profiles, including low smoking prevalence and moderate physical activity. Skinfold thickness was significantly greater in regular donors (*p* < 0.001). The main biological finding was a robust post-donation increase in HIF-1α concentrations (≈80%, *p* < 0.001), independent of donation frequency or lifestyle. No significant associations were found between lifestyle factors and vitamin B12 or folate levels. **Conclusions:** Blood donation induces a rapid elevation in HIF-1α, reflecting activation of hypoxia-responsive pathways and short-term hematopoietic adaptation. Beyond its biomedical relevance, voluntary blood donation represents a meaningful epidemiological and public health model for studying physiological resilience and the health benefits of altruistic behavior. These findings underscore the importance of donor surveillance and motivation as components of broader preventive health and health equity strategies.

## 1. Introduction

Blood and blood components are irreplaceable therapeutic resources in modern medicine, as they cannot be synthetically produced and rely exclusively on voluntary blood donors. The World Health Organization (WHO) identifies regular, unpaid voluntary donors as the safest source, reducing the risk of transfusion-transmissible infections and ensuring a reliable blood supply [1]. Blood transfusion, defined as the transfer of whole blood or components from donor to recipient [2], is indicated in acute or chronic blood loss, anemia, coagulation disorders, and as supportive therapy in malignant, hematologic, and chronic diseases [3,4,5]. Whenever possible, component therapy is preferred over whole blood, improving safety and cost-effectiveness [6]. Specific components (red blood cells, platelets, plasma, and cryoprecipitate) are administered according to pathophysiological needs to optimize efficacy and minimize adverse effects [7]. Beyond emergency medicine, transfusion plays a vital role in transplantation, oncology, neonatology, and rare hemostatic disorders [8].

Voluntary blood donation holds significant public health importance by preventing shortages, reducing transfusion-related mortality, and ensuring the timely availability of blood in emergencies [9]. Since transfusion medicine is integral to healthcare systems, the WHO recommends that blood collection, testing, processing, storage, and distribution be coordinated nationally within a regulated supply network, ensuring consistent safety and quality [10]. Achieving self-sufficiency through voluntary, unpaid donations is recognized as the most sustainable strategy and aligns with the WHO’s global vision of universal health coverage [11].

In Croatia, as elsewhere, blood supply depends heavily on voluntary donors, whose lifestyle and health behaviors influence both their own well-being and blood quality [12]. Understanding these determinants can improve donation practices and transfusion safety [13,14]. Regular donors undergo repeated examinations and screening [15,16,17], often benefiting from free health assessments and lifestyle counseling [18,19,20]. Blood donation also fosters solidarity and community engagement, promoting awareness of its importance [21].

Hematopoiesis, the process of blood cell formation, is regulated by nutrients (e.g., hematopoietic biomarkers vitamin B12 and folic acid), cytokines, and transcription factors [22]. Among them, hypoxia-inducible factor 1-alpha (HIF-1α) is a central regulator of hypoxic responses and hematopoiesis, supporting stem cell maintenance and erythropoiesis, while playing complex roles in hematologic malignancies. Modulation of HIF-1α offers therapeutic promise for anemia and certain cancers [23,24]. By activating genes involved in metabolism, angiogenesis, and erythropoiesis, HIF-1α mediates adaptation to oxygen deprivation [25], and its expression is linked to inflammatory, cardiovascular, and metabolic disorders, as well as physiological stressors such as blood donation [26].

Blood donation represents a controlled blood loss that stimulates hematopoiesis. Repeated donation has been shown to induce adaptive hematological changes, including erythropoietic regulation and altered iron metabolism [27], yet molecular mechanisms, particularly involving HIF-1α, remain insufficiently studied. Lifestyle factors such as smoking, physical activity, and diet further influence oxidative stress, inflammation, and hypoxic signaling [28,29]. Sociodemographic factors (age, sex, education, and motivation) also shape donation patterns, guiding public health interventions [30,31].

Although studied separately, the combined impact of sociodemographic, lifestyle, and biological factors on molecular pathways in healthy blood donors is poorly understood. Investigating HIF-1α expression in this context may provide insights into adaptive mechanisms of hematopoiesis. While donation is safe and routine, it constitutes a physiological stressor that can alter hypoxia-related molecular pathways. Current evidence on blood donation and HIF-1α expression is scarce, particularly regarding lifestyle influences.

This study aimed to examine the associations between blood donation and lifestyle factors with hematopoietic biomarkers (vitamin B12 and folic acid) and HIF-1α expression before and after donation, thus contributing to a better understanding of the biological effects of blood donation and their potential modulators.

## 2. Participants and Methods

### 2.1. Participants

A total of 324 voluntary blood donors were enrolled in the study and stratified into two groups based on their donation frequency: regular donors (study group) and occasional donors (control group). The sample size was calculated from the regional donor registry (*n* = 1399); ≥147 participants per group were required (power 80%, margin of error 5%, expected response 50%).

The study group comprised 159 active regular donors of both sexes, defined as individuals with a history of at least 20 lifetime donations and a consistent pattern of at least two donations per year over the preceding five years. This definition was chosen to ensure inclusion of participants with sustained and long-term exposure to repeated blood donation. Donors with irregular donation patterns were excluded from this group.

The control group included 165 healthy occasional donors, consisting of first-time donors or individuals who had not donated blood within the past year, thereby representing a population without regular exposure to donation. For both study and control groups, general exclusion criteria included the presence of acute or chronic illnesses, as well as any medical conditions or circumstances leading to temporary or permanent deferral according to standard blood donation eligibility guidelines. All participants were fully informed about the objectives and procedures of the study and provided written informed consent before enrollment.

### 2.2. Study Design

The research was conducted from July 2024 to February 2025 at the Clinical Institute of Transfusion Medicine, Clinical Hospital Centre Rijeka, Croatia, and during field campaigns. All participants underwent venipuncture for blood sampling, which was performed both before and after donation in order to measure serum concentrations of HIF-1α. Venous blood samples (5 mL) were collected at two time points: before donation (2.5 mL) and 30 min after blood donation (2.5 mL). Samples were centrifuged (2000 rpm, 10 min) and sera stored at −80 °C for further analyses. Following the initial blood sampling, participants completed three structured questionnaires and underwent skinfold measurements for body fat estimation. The questionnaires assessed socio-demographic characteristics, blood donation history, lifestyle factors, anthropometric data, and additional health-related information. Skinfold thickness was selected as a non-invasive, cost-effective, and widely validated method for estimating body fat percentage in population-based studies, offering reliable anthropometric data without the need for specialized imaging techniques. This multimodal data collection strategy enabled a comprehensive comparative analysis of the two donor groups by integrating biochemical markers, anthropometric measures, and self-reported information.

### 2.3. Questionnaires

#### 2.3.1. Sociodemographic Questionnaire

A sociodemographic questionnaire was specifically developed for the purposes of this study to capture a comprehensive profile of participants. The instrument consisted of a total of 19 items, 18 of which were structured, closed-ended questions designed to elicit standardized responses across key domains (e.g., age, sex, education, occupation, and socioeconomic indicators). In addition, one item was formulated as an open-ended question to allow participants to provide a written response in their own words. This latter component was intended to capture qualitative insights, thereby complementing the quantitative data obtained from the structured items.

#### 2.3.2. European Health Interview Survey (EHIS)

The European Health Interview Survey (EHIS) is a key tool developed to determine the European Core Health Indicators (ECHI), which were jointly developed by EU Member States and international organizations, taking into account the requirements of scientific and health policies. The indicators provide a framework for European health reporting, population health research and analysis, as well as the provision of healthcare at both the European and national levels [32].

Eurostat and external experts from EU Member States have developed a comprehensive manual containing detailed guidelines, for example, regarding the translation process and question sequence. It also proposes questions with response categories for each target variable, as well as details on sampling, weighting, and other technical aspects of the survey. The Commission Implementing Regulation for EHIS 2, Article 6, requires that finalized, validated, and weighted microdata, together with quality-related reference metadata, must be submitted in accordance with the quality and validation rules set by Eurostat. The Research Data Centre of the Robert Koch Institute (RKI) coordinated the data management process for German Health Update (GEDA—*Gesundheit in Deutschland aktuell*) 2014/2015-EHIS. All validation rules provided by Eurostat were strictly followed and processed. The dataset was further checked using the provided validation tool, “EDIT”—a software designed to verify whether the dataset was properly cleaned. The final microdata file was submitted to Eurostat via the Electronic Data and Metadata Information System (EDAMIS) in June 2016 and was reviewed, approved, and certified by Eurostat. Metadata and quality reporting follow the standard template developed by Eurostat, which contains information on the quality of the data file and is accessible on the Eurostat website [33].

The conceptual guidelines and survey instructions are organized according to the questionnaire model hierarchy, comprising modules, submodules, and individual variables. For each submodule, a brief description and rationale are provided. Some general guidelines applying to the entire submodule are also included in some cases. The remaining guidelines apply to individual variables and follow the same structure [34].

Permission to use the EHIS questionnaire for other research is regulated by the European Commission’s document use policy and is implemented under Commission Decision 2011/833/EU of 12 December 2011 on the reuse of Commission documents (OJ L 330, 14.12.2011, p. 39). Unless otherwise stated, the use of this document is permitted under the Creative Commons Attribution 4.0 International license (CC-BY 4.0) (https://creativecommons.org/licenses/by/4.0/ accessed on 12 January 2025). This means that use is permitted provided appropriate credit is given and any modifications are indicated.

For this research, the EHIS 3 questionnaire for Croatia was used, specifically items 1.4.1.—physical activity/exercise, 1.4.4.—smoking, 1.4.5.—alcohol consumption, totaling 22 questions. This questionnaire model was designed for face-to-face interviews (interviewer-administered, including PAPI/paper-and-pencil interviews and CAPI/computer-assisted personal interviews). These adaptations (for example, instructions to the interviewer on questions where show cards should be used) may be introduced provided that the modified question fully captures the core concept(s) of the original model question, and that the resulting target variable fully corresponds to the measurement of the variable with the modified version of the model question. In our study, face-to-face interviewing was conducted [35].

#### 2.3.3. Food Frequency Questionnaire (FFQ)

The Food Frequency Questionnaire (FFQ) is a validated tool for food consumption frequency and is widely used in epidemiological and nutritional research. It is used to collect data on dietary habits and patterns of consumption of specific foods, containing a list of various food items such as fruits, vegetables, cereals, meat, dairy products, and others. For each item, respondents answer questions about the frequency of consumption over a given period of time [36,37].

The purpose of the FFQ is to assess dietary habits regarding food group consumption over a longer period, usually several months or years. It enables the collection of data on dietary patterns and their relationship with various health outcomes, including cardiovascular diseases, diabetes, cancer, and others. The main feature of the FFQ is that it assesses relative rather than absolute intake, i.e., it serves to classify respondents of adequate or inadequate intake. Some authors consider the FFQ the best choice for research on the relationship between diet and health regarding the intake of macronutrients and micronutrients. Participants included in this research independently completed the self-administered sociodemographic questionnaire and the FFQ. The FFQ questionnaire has been validated in the Croatian language [38,39].

### 2.4. Laboratory Analysis

Serum concentrations of HIF-1α, vitamin B12 and Folic acid were quantified at predefined time points using a commercially available Human HIF-1α, vitamin B12 and Folic acid ELISA kit, (MyBioSource, San Diego, CA, USA; local distributor: Jasika d.o.o., Zagreb, Croatia). All assays were carried out strictly in accordance with the manufacturer’s instructions to ensure reproducibility and reliability of the results. Sample measurements were conducted at the Department of Medical Chemistry, Biochemistry, and Clinical Chemistry, Faculty of Medicine, University of Rijeka, Croatia. Each sample was analyzed in duplicate to minimize intra-assay variability, and absorbance values were obtained using a microplate ELISA reader (BIO-TEK EL808IU, BioTek Instruments Inc., Winooski, VT, USA). The mean of duplicate readings was used for statistical analysis. For data handling, duplicate values showing a coefficient of variation (CV) greater than 15% were re-analyzed. Quality control procedures were employed throughout the process to monitor assay performance, including adherence to recommended incubation times, temperature conditions, and the use of appropriate positive and negative controls.

### 2.5. Statistical Analyses

For statistical data analysis, MedCalc version 23.2.6 (Mariakerke, Belgium) was used. Qualitative/Categorical variables are presented as absolute and relative frequencies. Differences between groups were analyzed using the Chi-square test, and differences within a group were calculated using the proportions *t*-test. The normality of the distribution of quantitative variables was tested using the Kolmogorov–Smirnov test. Data are presented as the median and interquartile range (IQR), as well as the range from the minimum to the maximum value. Age is presented as the median and age range. Differences between groups were calculated using the Mann–Whitney U test, and differences in dependent samples were calculated using the Wilcoxon signed-rank test. All statistical values were considered significant at *p* < 0.05.

### 2.6. Ethical Considerations

The study was conducted in accordance with the Declaration of Helsinki, the Nuremberg Code, and Good Clinical Practice guidelines. All participants provided written informed consent. Ethical approval was obtained from the Ethics Committees of the University of Rijeka Faculty of Medicine (class: 007-08/24-01/21, registry no.: 2170-1-42-04-36/1-24-3) and the Clinical Hospital Centre Rijeka (class: 003-05/24-1/18, registry no.: 2170-29-02/1-23-2).

## 3. Results

A total of 324 participants were enrolled and stratified into regular (study group) and occasional (control group) donors. We compared the groups across four domains: (1) socio-demographic characteristics (age, sex, marital status, education, employment), (2) blood donation-related features (lifetime donations, donation frequency, donation history), (3) lifestyle and anthropometric data (height, weight, triceps skinfold, smoking, alcohol use, diet, physical activity), and (4) laboratory parameters (hematological and biochemical indices).

### 3.1. Socio-Demographic Characteristics

All participants resided within the area served by the Clinical Institute of Transfusion Medicine at Clinical Hospital Centre Rijeka, which covers three counties: Primorje-Gorski Kotar, Istria, and Lika-Senj. Socio-demographic characteristics of the 324 participants included in the study are summarized in Table 1.

Participants came from 78 different municipalities, with the majority from Rijeka (*n* = 72, 22.2%). The median age of all participants was 40 years (range: 18–68 years). Donors in the study group were significantly older than those in the control group (median age: 45 vs. 37 years; Mann–Whitney U test, Z = −5.66, *p* < 0.001). Regarding sex distribution, both groups were predominantly male (overall: 80.9% male vs. 19.1% female; *p* < 0.001). Within the study group, 91.8% (146/159) of participants were male and 8.2% (13/159) were female, whereas in the control group, 70.3% (116/165) were male and 29.7% (49/165) were female. This difference in sex distribution between groups was statistically significant (*p* < 0.001. These findings indicate that regular donors tended to be older and more frequently male compared with occasional donors, reflecting demographic patterns commonly reported in blood donor populations.

The majority of participants had completed secondary education (62.3%), while 25.6% held a university degree; the remaining participants had completed primary education or vocational training. No statistically significant differences in educational attainment were observed between the study and control groups (all *p* > 0.05), indicating a comparable distribution of education levels across donor categories (Table 1). Most participants were employed (84.3%), whereas a small proportion were unemployed (3.1%); the remainder reported other occupational statuses, including students and retirees. Employment status did not differ significantly between groups (all *p* > 0.05).

Analysis of personal monthly income showed that the largest proportion of participants belonged to the middle-income category (€1327.23–€1990.84; 37.7%), with smaller proportions reporting higher (>€1990.84; 13.9%) or lower (<€663.61; 9.0%) income levels. No significant differences in personal income distribution were found between the study and control groups (*p* > 0.05). Similarly, household income per capita was most frequently in the middle range (€929.06–€1327.23; 41.4%), followed by the higher-income category (>€1327.23; 23.8%), while only 2.2% of participants fell into the lowest-income group (<€265.44). Household income per capita was comparable between groups, with no statistically significant differences detected (*p* > 0.05). Overall, these findings indicate that the study and control groups were socioeconomically similar, minimizing the potential for income-related confounding. Nearly half of the participants were married (48.8%), whereas widowed individuals represented the smallest proportion of the study population (0.9%). No statistically significant differences in marital status distribution were observed between the study and control groups (all *p* > 0.05) (Table 1).

### 3.2. Blood Donation Characteristics

The median total number of lifetime blood donations among all participants was 18 (interquartile range [IQR]: 7–38; range: 0–135). As expected, regular donors exhibited a significantly higher number of donations (median: 37; IQR: 23–61) compared with occasional donors in the control group (median: 7; IQR: 4–13; *p* < 0.001). The overall median age at first blood donation was 20 years. Notably, individuals in the control group donated for the first time at a significantly older age compared with regular donors (median: 23 vs. 20 years; *p* < 0.001). A significantly larger proportion of donors (58.3%; *n* = 189; *p* = 0.003) reported having no family members who also donate blood, while 41.7% (*n* = 135) reported multiple family members as donors. In the study group, 42.0% had family members involved in blood donation, with a similar proportion in the control group (41.2%; *p* = 0.867).

In the total sample, the most prevalent blood group was O^+^ (33.3%), followed by A^+^ (29.9%) and B^+^ (17.6%). Blood group distribution did not differ significantly between the study and control groups (*p* = 0.511). Sex-stratified analyses showed a similar pattern: among men, O^+^ was the most common blood group (34.4%), followed by A^+^ (27.9%) and B^+^ (18.7%), with no significant difference between groups (*p* = 0.572). Among women, A+ predominated (38.7%), followed by O^+^ (29.0%) and B^+^ (12.9%), with no significant difference between the study and control groups (*p* = 0.978).

Regular donors demonstrated a markedly higher level of donation activity compared with occasional donors. The median lifetime number of blood donations in the regular donor group was 37 (IQR 23–61), whereas occasional donors reported a median of 7 donations (IQR 4–13), representing a statistically significant difference (*p* < 0.001). In addition, regular donors began donating blood at a significantly younger age than occasional donors, with a median age at first donation of 20 years (IQR 18–24) compared with 23 years (IQR 19–35) in the control group (*p* < 0.001), suggesting earlier engagement and longer donation careers among regular donors.

Nearly half of the overall study population (49.4%) reported having been deferred from blood donation at least once. The prevalence of previous deferral was slightly higher among regular donors (51.6%) than among occasional donors (48.4%); however, this difference was not statistically significant (*p* = 0.439). Similarly, the proportion of donors who had never experienced deferral was comparable between the two groups. These findings indicate that the likelihood of experiencing either temporary or permanent deferral was similar in regular and occasional donors, suggesting that deferral-related factors were not systematically associated with donation frequency in this cohort.

Low hemoglobin was the most frequent cause of blood donor deferral, accounting for more than half of all cases (54.4%). This was followed by combined low hemoglobin and blood pressure abnormalities (8.1%) and blood pressure abnormalities alone (7.5%). All other deferral reasons occurred considerably less frequently, each representing 5% or fewer cases. These included acute infections (such as common cold, herpes, mononucleosis, COVID-19, and hantavirus infection), recent medical or diagnostic procedures (including surgery, endoscopy, colonoscopy, and dental procedures), recent travel abroad, dermatological conditions, antibiotic use, elevated body temperature, cardiovascular findings, fatigue, inflammatory markers, and other isolated temporary conditions. The detailed distribution of all deferral causes is presented in Table 2. 

Deferrals occurred more frequently among women than men: 40 of 62 female donors (65%) were deferred compared with 120 of 262 male donors (46%). This difference was statistically significant (*p* = 0.038), indicating a higher deferral risk among female donors. Among deferred donors, men constituted the majority overall (75%), reflecting their higher representation in the study population. When stratified by donation frequency, the sex distribution of deferred donors differed between regular and occasional donors. In the regular donor group, most deferred individuals were men (90%), whereas in the occasional donor group, women accounted for a larger proportion of deferred donors (41%). However, the difference in sex distribution between regular and occasional donors within the deferred subgroup did not reach statistical significance for women (*p* = 0.104).

### 3.3. Nutritional and Lifestyle Habits

Dietary intake was assessed using a semi-quantitative frequency scale ranging from 1 (never or <1 time per month) to 6 (daily consumption). Overall, dietary patterns did not differ significantly between the experimental and control groups across the majority of assessed food categories. The sole exception was forest fruit consumption, which was reported at a significantly higher frequency in the control group compared with the experimental group (*p* = 0.025). This finding suggests a group-specific variation limited to this food category, while overall dietary intake profiles remained largely comparable (Supplementary Table S1).

According to data obtained from the European Health Interview Survey (EHIS), 50.3% of participants reported engaging in moderate-intensity physical activity during working hours, whereas 34.6% indicated that their occupational activities predominantly involved sitting or standing for the majority of the workday. In terms of active transportation, nearly half of the sample (49.1%) reported walking for at least 10 min per day as part of their commuting routine. Participation in recreational or sports-related physical activity was relatively limited, with a median frequency of 2–3 days per week, corresponding to an average of 2.5 h per week. When sedentary behavior was examined in greater detail, the study group reported a slightly lower median daily sitting time (4 h/day) compared with the control group (5.5 h/day). However, this difference did not reach statistical significance, indicating broadly comparable sedentary patterns between groups.

The majority of participants were non-daily smokers, indicating generally low levels of tobacco use within the study population. Quantitative assessment revealed a median cigarette consumption of 0 cigarettes per day (interquartile range [IQR]: 0–7), and a median smoking duration of 0 years (IQR: 0–10), reflecting a substantial proportion of participants who had never smoked or smoked only occasionally. Comparative analyses between the study and control groups indicated no statistically significant differences in either the intensity or duration of smoking, suggesting that tobacco use patterns were broadly similar across groups. These findings are consistent with a low overall prevalence of regular smoking in the cohort, which may have implications for associated cardiometabolic risk profiles and public health interventions.

Finally, anthropometric characteristics of the study population were assessed, revealing a median height of 180 cm (interquartile range [IQR]: 173–185 cm) and a median body weight of 90 kg (IQR: 80–101.5 kg). While overall body size did not differ markedly between groups, skinfold thickness (a proxy for subcutaneous adiposity) was significantly greater in the study group compared with the control group (*p* < 0.001). This finding suggests a higher accumulation of peripheral fat in the study group, despite similar overall body mass, highlighting potential differences in body composition that may not be captured by weight and height alone. These differences in adiposity could have important implications for metabolic risk and cardiometabolic health within the cohort.

### 3.4. Serum Hypoxia Inducible Factor 1 Concentrations

**Figure 1:**
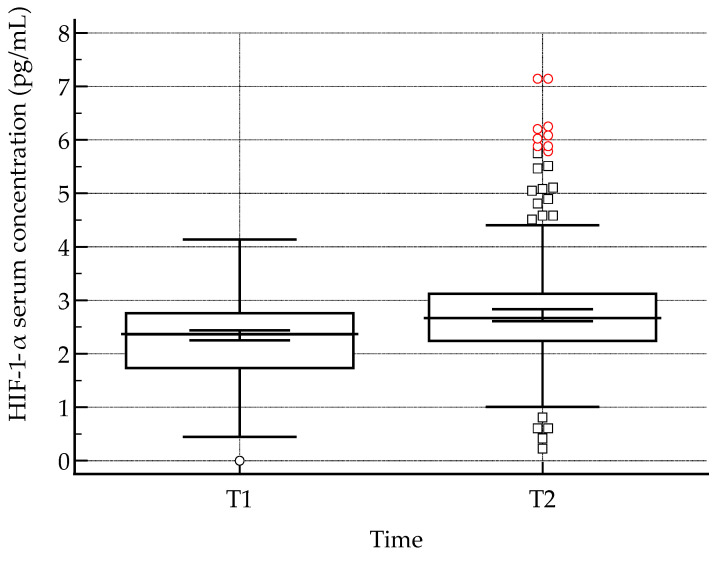
Serum levels of hypoxia-inducible factor 1-alpha (HIF-1α) were quantified at two time points: immediately before blood donation (HIF-1α, T1) and 30 min post-donation (HIF-1α, T2). Central lines represent the median; box limits indicate the interquartile range (IQR); and whiskers denote the full data range. Black circles and squares represent individual data points outside the range, while red dots indicate outliers.

Serum levels of hypoxia-inducible factor 1-alpha (HIF-1α) were quantified at two time points: immediately before blood donation (HIF-1α (T1)) and 30 min post-donation (HIF-1α (T2)). Across both the study and control groups, post-donation HIF-1α concentrations were consistently elevated relative to baseline measurements. Statistical analysis using the Wilcoxon signed-rank test confirmed that this increase was highly significant (*p* < 0.001), indicating a robust acute rise in HIF-1α levels following blood donation (Figure 1). These findings suggest that the donation procedure induces a measurable hypoxic or stress-related response, as reflected by the activation of HIF-1α, which may have downstream effects on cellular adaptation, oxygen homeostasis, and erythropoietic signaling.

Changes in serum HIF-1α levels were assessed longitudinally, with values expressed relative to baseline (pre-donation) measurements. This approach allowed for the evaluation of dynamic alterations in HIF-1α expression induced by the donation procedure. Serum HIF-1α concentrations increased markedly following blood donation. Mean HIF-1α levels rose from 1.0 ± 0.2 pg/mL before donation to 1.8 ± 0.3 pg/mL after donation, representing an approximate 80% increase relative to baseline. Pre-donation values ranged from 0.7 to 1.3 pg/mL, while post-donation concentrations ranged from 1.4 to 2.3 pg/mL, indicating a consistent elevation across participants. The relative expression data illustrate the magnitude and direction of post-donation changes, providing insight into the acute hypoxic and stress-related responses triggered by blood loss. Monitoring HIF-1α trends in this manner enables a clearer understanding of the temporal activation of hypoxia-responsive pathways and their potential physiological consequences.

### 3.5. Serum Levels of Folic Acid and Vitamin B12

Serum concentrations of folic acid and vitamin B12 were assessed in 311 participants to evaluate micronutrient status. Both biomarkers showed marked interindividual variability. Folic acid concentrations ranged from 225.7 to 15,169.5 pg/mL, with a mean value of 4630.1 pg/mL and a median of 2578.1 pg/mL. Vitamin B12 concentrations ranged from 8.9 to 820.3 pg/mL, with a mean of 141.2 pg/mL and a median of 132.0 pg/mL. The interquartile range was wide for both analytes (folic acid: 1558.3–7552.1 pg/mL; vitamin B12: 105.7–164.3 pg/mL), reflecting substantial dispersion within the cohort. Distributions of both folic acid and vitamin B12 concentrations deviated significantly from normality, as confirmed by the Shapiro–Wilk test (*p* < 0.0001 for both), indicating skewed distributions and supporting the use of nonparametric statistical methods in subsequent analyses.

Comparison of serum folic acid concentrations between the study group and the control group revealed that median folic acid levels were lower in the study cohort (2287.6 pg/mL) relative to the control group (4453.1 pg/mL). Despite this apparent difference, the observed variation did not achieve statistical significance (*p* = 0.192), indicating that, within the current sample, folic acid levels were not significantly altered between groups.

**Figure 2:**
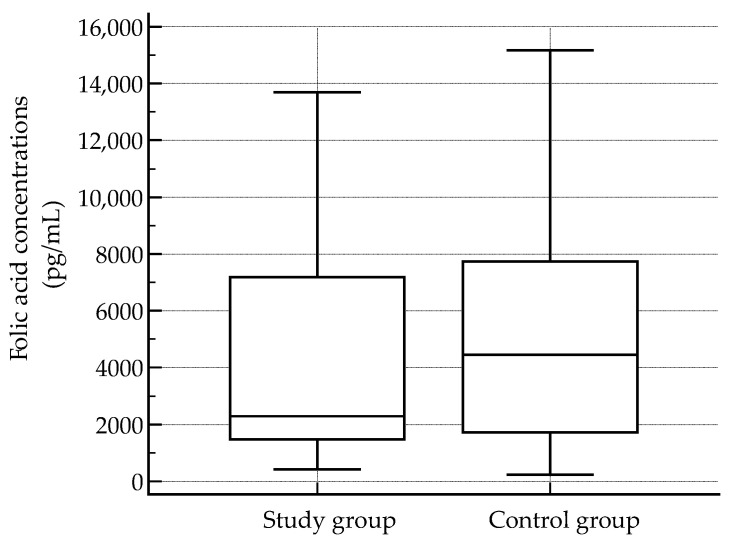
Serum folic acid concentrations. Central lines represent the median; box limits indicate the interquartile range (IQR); and whiskers denote the full data range.

Serum folic acid concentrations showed wide variability in both groups. In the study group (*n* = 152), folic acid values ranged from 421.4 to 13,694.9 pg/mL, with a median concentration of 2287.6 pg/mL. In the control group (*n*= 159), concentrations ranged from 225.7 to 15,169.5 pg/mL, with a higher median value of 4453.1 pg/mL. The interquartile ranges overlapped substantially between groups, indicating considerable dispersion and variability in folic acid concentrations across both donor categories (Figure 2).

**Figure 3:**
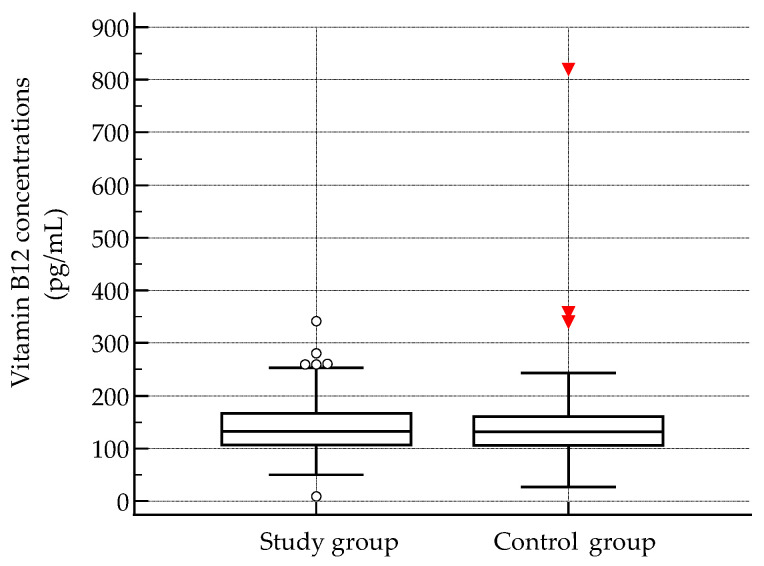
Serum vitamin B12 concentrations. Central lines represent the median; box limits indicate the interquartile range (IQR); and whiskers denote the full data range. Black circles represent individual data points outside the range, while red triangles indicate outliers.

Serum vitamin B12 concentrations were highly comparable between the study and control groups, with median values of 132.2 pg/mL and 131.8 pg/mL, respectively. Statistical analysis confirmed that this minimal difference was not significant (*p* = 0.761), indicating that vitamin B12 status was essentially equivalent across the two cohorts (Figure 3) 

## 4. Discussion

This study aimed to examine the associations between socio-demographic characteristics, lifestyle habits, and anthropometric measures of voluntary blood donors, and the expression of the transcription factor HIF-1α before and after blood donation, as well as the serum levels of hematopoietic biomarkers vitamin B12 and folic acid. The findings provide insight into both the profile of voluntary blood donors and the biological mechanisms underlying adaptation to blood donation as a form of controlled blood loss.

In our study, men constituted the majority of donors (80.9%), which is consistent with previous studies showing male predominance among blood donors in Europe and worldwide [40,41]. Several factors likely contribute to this pattern. Physiologically, women face certain limitations, including lower hemoglobin levels due to iron-deficiency anemia, pregnancy or lactation, more frequent temporary deferrals, restrictions during menstruation, and longer legally mandated donation intervals (four months for women versus three months for men) [42]. Social and cultural influences may further shape donation behaviors. Within our study group, 91.8% of donors were male compared with 70.3% in the control group, suggesting that men not only donate more frequently but also establish a more regular donation habit. Age analysis revealed that regular donors were older, with a median age of 45 years compared to 37 years for occasional donors, indicating that sustained donation habits develop over time and that older donors demonstrate greater persistence and motivation, supporting previous observations [42,43,44]. Educational level, employment status, and income did not differ significantly between groups, with most donors having completed secondary education, being employed, and belonging to middle-income brackets. These results suggest that blood donation occurs across socio-economic strata, without a clear association with educational or financial status, mirroring trends observed in other European populations [40,41,42,43,44,45].

Both vitamin B12 and folic acid are essential for erythropoiesis and DNA synthesis, and their adequate levels are important for normal hematopoietic response after blood loss. Although in our study we did not find statistically significant differences between regular and occasional donors, the observed variability indicates that individual nutritional habits and absorption may still play a role. Different studies have shown that low levels of these vitamins are associated with impaired mitochondrial function and oxidative stress, while folic acid contributes to nitric oxide regulation and vascular response to hypoxia [23,24,25,26,27,28,29]. The observed absence of statistically significant differences in the content of folic acid in our research may be attributable to interindividual variability, limited sample size, or the influence of unaccounted confounding factors affecting folic acid status. On the other hand, the observed similarity in B12 concentrations indicates that, in contrast to folic acid, vitamin B12 levels were not influenced by the conditions or interventions characterizing the study population. Insufficient folic acid or vitamin B12 can lead to slower regeneration of red blood cells and reduced tissue oxygenation, which could, at least theoretically, affect HIF-1α activation and the overall adaptive response to donation. Some experimental studies have shown that low levels of these vitamins are associated with impaired mitochondrial function and oxidative stress, while folic acid contributes to nitric oxide regulation and vascular response to hypoxia. Considering that most donors in our sample probably had adequate nutritional status, this could explain why HIF-1α increased consistently across both groups, regardless of donation frequency or lifestyle factors. Nevertheless, monitoring folic acid and vitamin B12 in donor populations remains important, especially in older or female donors who are more prone to subtle deficiencies that might influence hematopoietic recovery over time.

Analysis of blood donation characteristics revealed that regular donors reported a substantially higher number of lifetime donations, with a median of 37 compared with seven for occasional donors, and they began donating at a younger age (20 versus 23 years). Early involvement in donation appears to foster the development of long-term donation habits [42,43], highlighting the importance of programs targeting youth, such as school-based blood drives in Primorje-Gorski Kotar County organized by the Red Cross and the Clinical Institute of Transfusion Medicine. Expanding and systematizing such initiatives could improve recruitment and retention of new donors. Low hemoglobin was the most frequent reason for temporary deferral (54.4%), consistent with previous research identifying iron-deficiency anemia as the primary cause [46]. Women were more often deferred, reflecting known sex differences in iron stores [46,47]. Evidence from a Dutch stepped-wedge cluster-randomized trial (FIND’EM) demonstrated that ferritin-guided donation intervals significantly improved hemoglobin and ferritin levels and reduced the prevalence of iron deficiency in whole-blood donors [48]. Furthermore, a study of donor behavior following ferritin-based deferrals showed that while iron status improved, donor return rates were modestly affected, underscoring the need for parallel retention strategies [49]. These findings are in line with broader evidence that individualized donation policies can protect donor health and sustain donation frequency [50]. Implementing similar strategies in the Croatian system could particularly benefit female donors and support the sustainability of the donor pool.

Results of lifestyle and anthropometric characteristics, dietary habits were broadly similar between regular and occasional donors, except slightly higher forest fruit consumption in the control group. Overall, donor diets reflected those of the general Croatian population, without clear trends toward healthier or less healthy patterns. Since diet can influence oxidative stress and the regulation of molecules such as HIF-1α [47,51], these results suggest that dietary habits were not a primary driver of the observed biological response to blood donation. Physical activity levels were moderate across both groups, and smoking prevalence was low, with no significant differences detected. These findings indicate that blood donors generally do not exhibit high-risk lifestyle behaviors, consistent with previous reports highlighting healthier behaviors among donors compared with the general population [40,45,51]. Taken together, these findings suggest that while a substantial proportion of participants achieve moderate physical activity in occupational and commuting contexts, engagement in structured leisure-time activity remains suboptimal, and sedentary behavior persists at levels of potential public health concern. Anthropometric assessment revealed significantly greater skinfold thickness among regular donors, although height and weight did not differ between groups. This difference may reflect variations in fat distribution or metabolic characteristics, though further research is warranted given potential measurement variability.

A central finding of the study was a marked increase in HIF-1α levels following blood donation in both regular and occasional donors, supporting the concept that controlled blood loss acts as a physiological stressor, eliciting adaptive responses related to hypoxia and hematopoiesis [52,53,54]. HIF-1α is a critical transcription factor regulating the cellular response to low oxygen levels, stimulating erythropoiesis and angiogenesis [53,54,55]. It modulates the expression of over 60 genes, including erythropoietin and vascular endothelial growth factor, which directly influence red blood cell production, vascular development, and iron metabolism [56]. These results align with prior studies demonstrating that blood loss can modulate transcription factor activity and subsequently affect hematopoiesis [46,57,58]. Notably, the observed post-donation increase in HIF-1α occurred regardless of donation frequency, suggesting a general physiological response to acute blood loss. To the best of our knowledge, this study is the first to investigate the dynamic changes in circulating HIF-1α levels in voluntary blood donors, including measurements taken immediately before and right after donation, using an integrated epidemiological and biochemical approach. Although no previous work has examined this response in the context of blood loss or hemodilution, findings from other hypoxia-related research help place our observations into perspective. In experimental and clinical settings, HIF-1α is known to stabilize very rapidly when oxygen availability decreases, with measurable increases reported within minutes to the first hour of hypoxic exposure. These early changes in protein stabilization occur well before downstream transcriptional effects, which typically peak several hours later. Such evidence suggests that acute physiological events capable of transiently reducing oxygen delivery may trigger similarly fast HIF-1α responses. By capturing measurements at the earliest feasible time points surrounding blood donation, our study provides new insight into how promptly HIF-1α may respond in vivo under real-world conditions, offering a valuable contribution to the limited literature on temporal HIF-1α dynamics in humans [59,60,61]. By combining molecular analysis with detailed sociodemographic and lifestyle profiling, this research provides a novel perspective on adaptive physiological mechanisms in a rigorously screened, healthy population. The limited existing literature specifically linking blood donation to HIF-1α expression underscores the novelty of these findings. Future research should incorporate long-term follow-up to determine whether regular donors exhibit distinct patterns of HIF-1α adaptation over months and to further elucidate the mechanisms underlying donor resilience and recovery.

The study offers several important implications. First, it confirms that voluntary blood donors represent a population with generally healthier lifestyle habits and a predominance of males [40,41]. Second, the observed rise in HIF-1α after donation highlights the potential for further investigation into its role in hematopoietic adaptation and resilience to hypoxia [53,54,55,59,60,61]. Third, our findings underscore the need to support female donors, particularly regarding nutritional status and anemia prevention, as demonstrated by recent European studies. Overall, these results emphasize the importance of evidence-based public health strategies for blood donation, including targeted recruitment of young healthy donors to maintain a stable and safe blood supply [46,48,49].

### Study Limitations

Despite several strengths, including a relatively large and well-characterized sample and the integration of both biological measurements and detailed survey data, this study has several limitations that warrant consideration. First, the cross-sectional design precludes the ability to draw conclusions regarding the long-term effects of repeated blood donation on HIF-1α expression and related physiological adaptations. Longitudinal studies are needed to determine whether cumulative donations alter baseline or post-donation HIF-1α dynamics and associated hematopoietic and vascular responses. Second, lifestyle and dietary information was obtained via self-reported questionnaires, which may be subject to recall bias, social desirability bias, or inaccurate reporting, potentially affecting the precision of associations between behavioral factors and HIF-1α levels. Third, although validated instruments and standardized laboratory procedures were employed to minimize measurement error, some variability in anthropometric, biochemical, or transcription factor assessments cannot be entirely excluded. Finally, while the study provides novel insights into HIF-1α responses in blood donors, the generalizability of findings may be limited to similar donor populations and may not extend to other demographic or ethnic groups. Acknowledging these limitations underscores the need for follow-up studies with longitudinal designs, objective lifestyle monitoring, and broader population sampling to confirm and extend these findings.

Future research should employ longitudinal designs with repeated HIF-1α measurements at multiple time points after donation to better capture the temporal dynamics of the adaptive response. Investigating associations between HIF-1α, iron status, erythropoietin, and oxidative stress markers could further clarify mechanisms of hematopoietic regulation [46,47,53,54,55,56,57,58,59,60,61]. Additionally, the effects of lifestyle factors such as antioxidant-rich diets or physical activity on HIF-1α expression and donor health warrant further exploration [47,51,59,60,61].

## 5. Conclusions

In summary, this study demonstrates that voluntary blood donors exhibit distinct socio-demographic and behavioral profiles, characterized by male predominance, higher age among regular donors, and generally moderate-risk lifestyle habits. Early engagement in donation appears to foster long-term retention, highlighting the importance of youth-focused recruitment and education programs. Blood donation induces a robust and consistent increase in HIF-1α levels, reflecting an acute physiological response to controlled blood loss. Mechanistically, HIF-1α acts as a key regulator of the cellular hypoxic response, stimulating erythropoiesis through upregulation of erythropoietin, promoting angiogenesis via vascular endothelial growth factor, and modulating iron metabolism and oxygen delivery pathways. These transcriptional effects support rapid adaptation to reduced oxygen availability and facilitate hematopoietic recovery. While general lifestyle factors such as diet, physical activity, and smoking appear to exert limited influence on this acute response, variations in body composition, particularly subcutaneous adiposity, may modulate HIF-1α dynamics and downstream hematopoietic and vascular processes. Collectively, these findings highlight the potential of HIF-1α as a biomarker for donor resilience and recovery, provide mechanistic insight into the physiological adaptations triggered by blood donation, and inform strategies to optimize donation intervals, iron supplementation, and donor health monitoring. These insights could support the development of evidence-based donor management and nutritional monitoring programs within national transfusion services. Future longitudinal studies are warranted to evaluate cumulative effects of repeated donation on HIF-1α-mediated pathways, erythropoiesis, and vascular adaptation, ultimately enhancing both donor safety and the efficiency of blood collection systems. These findings highlight the importance of ongoing donor health monitoring and encourage further research into the molecular responses associated with blood donation.

## Figures and Tables

**Table 1:** Socio-demographic characteristics of the 324 participants included in the study.

Characteristic	Study Group (*n* = 159)	Control Group (*n* = 165)	Total (*n*= 324)	*p*-Value
Age, median (range)	45 (22–68)	37 (18–62)	40 (18–68)	<0.001
Sex (M/F)	146/13	116/49	262/62	<0.001
Education				
Primary school	3 (1.9%)	3 (1.8%)	6 (2.0%)	0.993
High school	109 (68.6%)	93 (56.4%)	202 (62.3%)	0.074
College	15 (9.4%)	18 (10.9%)	33 (10.2%)	0.889
University	32 (20.1%)	51 (30.9%)	83 (25.6%)	0.282
Employment status				
Employed	138 (86.8%)	135 (81.8%)	273 (84.3%)	0.257
Unemployed	2 (1.3%)	8 (4.8%)	10 (3.1%)	0.832
Student	1 (0.6%)	20 (12.1%)	21 (6.5%)	0.732
Retired	18 (11.3%)	2 (1.2%)	20 (6.2%)	0.664
Marital status				
Single	29 (18.2%)	52 (31.5%)	81 (25.0%)	0.198
Married	96 (60.4%)	62 (37.6%)	158 (48.8%)	0.0053
Common-law union	22 (13.8%)	41 (24.8%)	63 (19.4%)	0.310
Divorced	10 (6.3%)	9 (5.5%)	19 (5.9%)	0.942
Widowed	2 (1.3%)	1 (0.6%)	3 (0.9%)	0.964

**Table 2:** Causes of Blood Donor Deferral.

Reasons for Deferral	*n*	%
Low hemoglobin	87	54.4
Blood pressure abnormalities and low hemoglobin	13	8.1
High or low blood pressure	12	7.5
Common cold	8	5.0
Recent surgery	7	4.4
Herpes infection	4	2.6
Travel abroad	4	2.6
Dental procedure	3	1.9
Colonoscopy	2	1.3
Mononucleosis	2	1.3
Elevated body temperature	2	1.3
Acupuncture as pain therapy	1	0.6
Acute illness	1	0.6
Antibiotic use	1	0.6
COVID-19	1	0.6
Dermatitis of the face	1	0.6
Endoscopy within the past 4 months	1	0.6
Excess supply of the donor’s blood group	1	0.6
Fatigue	1	0.6
Fracture	1	0.6
High heart rate (tachycardia)	1	0.6
Increased inflammatory markers	1	0.6
Recent tattoo	1	0.6
Rash	1	0.6
Recent cut or wound	1	0.6
Hantavirus infection	1	0.6
Ongoing medical therapy	1	0.6
Total	160	100

## Data Availability

The raw data supporting the conclusions of this article will be made available by the authors on request.

## Supplementary Materials

The following supporting information can be downloaded at: https://www.mdpi.com/article/10.3390/epidemiologia7010009/s1, Table S1: Participants’ dietary habits.
