# Supplementary material for: Sociodemographic and Lifestyle Determinants of HIF-1α Response to Blood Donation and Hematopoietic Factors: Epidemiological and Public Health Perspectives from Voluntary Donors
Source: Epidemiologia (Basel). 2026 Jan 5;7(1):9. doi: 10.3390/epidemiologia7010009 (PMC12821670; doi:10.3390/epidemiologia7010009)
Supplement: Supplementary file 1 [file epidemiologia-07-00009-s001.zip › epidemiologia-3985458 - supplementary.pdf]

epidemiologia

MDPI

Table S1. Participants' dietary habits.

Food Item	Study Group (n = 159)	Control Group (n = 165)	Total (n = 324)	p
Red meat	4 (3–4)	4 (3–4)	4 (3–4)	➢ 0.05
White meat	4 (3–4)	4 (3–4)	4 (3–4)	➢ 0.05
Processed meat products	4 (3–4)	4 (3–4)	4 (3–4)	➢ 0.05
Eggs	4 (3–4)	4 (3–4)	4 (3–4)	➢ 0.05
Oily fish	2 (2–3)	2 (2–3)	2 (2–3)	➢ 0.05
White fish	2 (2–3)	2 (1.75–3)	2 (2–3)	➢ 0.05
Milk	4 (3–6)	3 (3–6)	4 (3–6)	➢ 0.05
Yogurt/kefir	4 (3–4)	4 (3–4)	4 (3–4)	➢ 0.05
Cheese	4 (3–4)	4 (3–5)	4 (3–4)	➢ 0.05
Butter	3 (2–3)	3 (2–4)	3 (2–4)	➢ 0.05
Cream	2 (1–3)	2 (1–3)	2 (1–3)	➢ 0.05
Green leafy vegetables	4 (3–4)	4 (3–5)	4 (3–4,5)	➢ 0.05
Other vegetables	4 (3–4)	4 (3–4)	4 (3–4)	➢ 0.05
Potato/sweet potato	4 (4–5)	4 (4)	4 (4–5)	➢ 0.05
Root vegetables	4 (3–4)	4 (3–4)	4 (3–4)	➢ 0.05
Fruit	4 (4–5)	4 (3–5)	4 (3–5)	➢ 0.05
Citrus fruit	4 (3–4)	4 (3–5)	4 (3–5)	➢ 0.05
Forrest fruits	2 (2–3)	3 (2–4)	2.5 (2–4)	0.025
Nuts	3 (2–4)	3 (2–4)	3 (2–4)	➢ 0.05
Avocado	1 (1)	1 (1)	1 (1)	➢ 0.05
Legumes	3 (2–4)	3 (2–4)	3 (2–4)	➢ 0.05
Cereals	3 (2–4)	3 (2–4)	3 (2–4)	➢ 0.05

 https://doi.org/10.3390/epidemiologia7010009
